# A continental-wide decline of occupancy and diversity in five Neotropical carnivores

**DOI:** 10.1016/j.gecco.2024.e03226

**Published:** 2024-11

**Authors:** Florencia Grattarola, Kateřina Tschernosterová, Petr Keil

**Affiliations:** Faculty of Environmental Sciences, Czech University of Life Sciences Prague, Kamýcká 129, Praha – Suchdol 16500, Czech Republic

**Keywords:** AOO, Biodiversity change, Geographic range, Dynamic patterns, Hotspots of change, Integrated species distribution models, Bayesian, Species richness

## Abstract

The Neotropics are a global biodiversity hotspot that has undergone dramatic land use changes over the last decades. However, a temporal perspective on the continental-wide distributions of species in this region is still missing. To unveil it, we model the entire area of occupancy of five Neotropical carnivore species at two time periods (2000–2013 and 2014–2021) using integrated species distribution models (ISDMs) in a Bayesian framework. The carnivores are the jaguarundi (*Herpailurus yagouaroundi*), margay (*Leopardus wiedii*), maned wolf (*Chrysocyon brachyurus*), tayra (*Eira barbara*), and giant otter (*Pteronura brasiliensis*). We mapped the temporal change, the areas where gains and losses accumulated for all species (hotspots of change) and calculated the temporal species turnover and change in spatial turnover. We show that (1) most carnivore species have declined their area of occupancy (i.e., range size) in the last two decades, (2) their diversity has decreased over time, mostly in the Chaco region, and (3) that hotspots of fast species composition turnover are in Chaco, the Caatinga region, and northwest of Mexico. We discuss how these newly identified hotspots of change overlap with regions of well-known and pronounced land use transformation. These estimated patterns of overall decline are alarming, more so given that four out of the five species had been classified as not threatened by IUCN. The official global threat status of these species may need to be re-evaluated. All this would be invisible if standard forecasts, local expert knowledge, or static threat criteria, such as range size, were used. We thus provide a new approach to evaluate past species range dynamics based on multiple lines of evidence, which can be employed over more species in the future, particularly in under-sampled regions.

## 1 Introduction

The Neotropics are biologically megadiverse (Grenyer et al., 2006, Raven et al., 2020) but also face one of the most significant degradations of natural areas (Barlow et al., 2018; WWF, 2020). The main land transformations are related to converting native grasslands and forests into farming lands (e.g., soybean plantations), pastures for cattle ranching, and exotic-tree forestry (Baeza et al., 2022; Curtis et al., 2018; Pompeu et al., 2023; Song et al., 2021; Souza et al., 2020). Likely as a consequence, there have been reports of defaunation in the Neotropics (Bogoni et al., 2020, Emer et al., 2019, Magioli et al., 2021). While these studies have contributed valuable insights, they are either geographically limited in their extent (e.g., local to the Atlantic forest, Emer et al. (2019), or Caatinga, Moura et al., 2023b), temporally too broad (e.g., comparing current distributions with late Quaternary periods, Sandom et al., 2014), or they rely on forecasts or hindcasts rather than on direct empirical comparisons (e.g., using IUCN range maps, Bogoni et al., 2020). A data-driven study with a continental extent that aligns with the recent degradation of natural areas (i.e., last 20 years) is missing.

At the large continental extent, a fundamental property of a species is its geographical range (Brown et al., 1996): the set of limits to the spatial distribution of a species (e.g., boundaries within which a species occurs) (Gaston, 2003). We can stack ranges of multiple species to get continental maps of species diversity (Mittermeier et al., 2011, Myers et al., 2000, Roll et al., 2017), and we can use them to study the differentiation of species composition, the so-called beta diversity (Anderson et al., 2011; Koleff et al., 2003). A joint knowledge of these properties (i.e., species ranges, species diversity, and beta diversity) gives a holistic picture of the state of nature at large scales. While a map of species richness can show biodiversity hotspots, considering it jointly with the identity and area of occupancy of each species can reveal centres of endemism, as well as areas with unique species composition. In the Neotropics, at the resolution of 100 ×100 km, species ranges for vertebrates have long been available (Schipper et al., 2008) and analysed (Coelho et al., 2023). The problem is that all of this is completely static. We still don’t know how it all changes in time, even though the knowledge of the temporal dynamics of species’ occupancy (are species expanding or shrinking?), diversity (are sites losing or gaining species?), and beta diversity (is there biotic homogenisation or differentiation?) is potentially critical in the face of the ongoing land transformation in the Neotropics.

The main reason for the lack of knowledge about continental-wide temporal dynamics is the amount and quality of data in the region (Hortal et al., 2015). Datasets collected/observed at the same location at different points in time over large spatial extents, such as national gridded atlases, are practically non-existent. This is a cross-cutting issue, as it limits our capacity to address the biodiversity knowledge gap, identify threats to biodiversity, and take evidence-based decisions and actions.

Data about species distribution can come in different forms (Kissling et al., 2018), and these are the most commonly accessible in the Neotropics:

1. First, presence-only point occurrences are the most common type of observation data. They frequently originate from specimens deposited in scientific collections and opportunistic observations and are available through global aggregators such as GBIF (the Global Biodiversity Information Facility). Contributing these using smartphones through community science initiatives has become popular in the region (Pocock et al., 2018). iNaturalist, for instance, has nine national sites in the region (Mexico, Guatemala, Costa Rica, Panama, Colombia, Ecuador, Chile, Argentina, and Uruguay). The point occurrences that this platform hosts are of particular importance in such an under-sampled region, as they often cover a larger portion of area than structured survey data. However, like most incidental presence-only observations, they are usually spatially, temporally, and taxonomically biased (Boakes et al., 2010, Oliveira et al., 2016).
2. Second, high-quality biodiversity data, for which we know the exact sampling effort and methods and where both species’ presences and absences are recorded, are rarely shared through the GBIF initiative. Examples are data from camera traps (Steenweg et al., 2017), whose use in Latin America is increasing rapidly (Delisle et al., 2021). Despite previous efforts to collate camera trap surveys in the region (Ahumada et al., 2011), plenty of heterogeneous data still can be mobilised (Kühl et al., 2020, Scotson et al., 2017).
3. Third, expert range maps (IUCN, 2023) are a major data source for macroecological studies in this region. They represent aggregated expert knowledge to estimate the broad boundaries of areas where a species is expected to be found (Jetz et al., 2012). They are built using data on the species’ evolutionary history, habitat preferences, current occurrences and local knowledge. In many regions, such as the Neotropics, they can represent the only available knowledge of the distribution of a species. However, they only indicate the absence of a species beyond range boundaries, not its exact sites of presence within the range. Further, they contain large spatial and temporal uncertainties, which restricts their usefulness in tracking changes in species distributions over time.

A new approach to addressing the issues of data incompleteness and heterogeneity is using Integrated Species Distribution Models (ISDMs) (Isaac et al., 2020). ISDMs usually assume that the true but unobserved distribution of a species (the spatial locations of individuals) can be modelled by a Poisson point process conditional on, for instance, environmental covariates. This true distribution can then be sampled through different observation processes, generating the data we observe (presence-absence, presence-only, or abundance). The parameters affecting the intensity of the resulting point pattern can then be estimated using the joint likelihood for the different data types while accounting for the specific ways they were observed. Thus, ISDMs take advantage of the strengths of different data types, and they can also explicitly account for their limitations, like imperfect detection, sampling bias, uneven effort, and varying survey areas (Fletcher Jr. et al., 2019; Miller et al., 2019; Pacifici et al., 2017). Most ISDMs implementations are fitted in a Bayesian framework, which has the advantage of propagating the uncertainties associated with each data type into the predictions and parameter estimates. However, using ISDMs to statistically integrate different types of data together (e.g., presence-only, presence-absences, range maps) and assess the range dynamics of multiple species at continental scales in regions with limited data availability is still rare (e.g., Grattarola et al., 2023).

A key taxonomic group to explore range dynamics in the Neotropics is the Carnivores (Mammalia: Carnivora). This group includes some of the most charismatic and well-known mammalian species (Valkenburgh, 1999), such as the jaguar (*Panthera onca*) and the Andean bear (*Tremarctos ornatus*). These and other species represent the higher trophic levels within the Neotropics and thus have a key role in ecosystem functioning here (e.g., through predation of herbivores and intraguild competition; Metz et al., 2024; Ripple et al., 2014; Roemer et al., 2009). Carnivores are also subjects of human-wildlife conflicts in the region (e.g., Castaño-Uribe et al., 2017), which are likely to increase with the expansion of productive lands and urban areas over natural habitats (Pereira et al., 2024). Hence, working with this group can provide important information for assessing anthropogenic changes in megadiverse ecosystems, such as those occupied by these species in the Neotropics.

The aim of this study is to model temporal change in the geographic distribution of eight mammal carnivores over the entire Neotropics using a recently developed ISDM (Grattarola et al., 2023). We chose ISDMs because, unlike any other correlative SDM (e.g., MaxEnt), they allow us to integrate different data types (i.e., multiple lines of evidence), account for varying sample area, sampling intensity and spatial autocorrelation, and include a temporal dimension (Schank et al., 2017). We map the geographic range of species in two time periods, 2000–2013 and 2014–2021, quantify the changes in the area of occupancy (i.e., range size) of the species, and ask the following questions: i) how have species’ geographic ranges changed over time, contracted vs. expanded, where and in which direction? ii) how has the species diversity changed? (i.e., species richness) and iii) has dissimilarity among assemblages decreased (i.e., biotic homogenisation) or increased (i.e., biotic differentiation)?

## 2 Material and methods

We estimated changes in the geographic distribution of species using an ISDM that integrated camera trap survey data with GBIF point occurrences and IUCN range maps, and considered different covariates for each species and accounted for sampling effort and spatial autocorrelation. We used the ISDM to map the occupancy (i.e., probability of occurrence) for each species over time, calculated the changes in the areas of occupancy (i.e., range size), mapped temporal species turnover between the two time periods, and estimated the temporal change in spatial beta diversity.

### 2.1 Species data, observation effort and environmental predictors

The eight species included in the study are the jaguarundi *Herpailurus yagouaroundi* (É. Geoffroy Saint-Hilaire, 1803), ocelot *Leopardus pardalis* (Linnaeus, 1758), margay *Leopardus wiedii* (Schinz, 1821), coati *Nasua nasua* (Linnaeus, 1766), crab-eating fox *Cerdocyon thous* (Linnaeus, 1766), maned wolf *Chrysocyon brachyurus* (Illiger, 1815), tayra *Eira barbara* (Linnaeus, 1758), and giant otter *Pteronura brasiliensis* (E. A. W. Zimmermann, 1780). We chose these species because they had sufficient data (i.e., more than 5 records per time period) and no taxonomic issues (e.g., recent taxonomic revision) and to have a balanced representation of the Carnivore biota as they fall under different conservation categories and distribute from south to north of the Neotropics. Based on model performance, we failed to model three of these species (coati, crab-eating fox, and ocelot) and thus they are not included in the posterior analyses (see Results).

Since we aimed to compare the distributional change in time, we divided the data into two time periods (time1: 2000–2013 and time2: 2014–2021), the minimum possible to see changes in time given the low number of data points we had for some species (Table 1). The temporal span was chosen considering most of the data available were collected from 2000 onwards. The temporal division was chosen to be able to represent, on average, 50 % of the data (presence-absence and presence-only) in each period. We expected to have similar uncertainties of distributions due to similar sample sizes in each time period while retaining sufficient data to produce the best estimate of the current distribution without having convergence issues.

**Table 1:** Species data. Presence-only (PO) and presence-absence data (PA), and covariates used for the eight carnivore species. For each data type (PO and PA), we show the total number of records included for each species and the number of records for each period (time1: 2000–2013, and time2: 2014–2021). The IUCN category is shown for each species (LC: least concern, NT: near threatened, EN: endangered). For an explanation of the covariates’ abbreviations, see Table A.1.

**Species**	**IUCN status**	**PO data**		**PA data**		**covariates**
		**total**	**time1 | time2**	**total**	**time1 | time2**	
*Herpailurus yagouaroundi***(jaguarundi)**	Least concern (LC)	**804**	216 | 588	**602**	290 | 312	npp, elevation, bio7, bio15
*Leopardus pardalis* ***(ocelot)***	Least concern (LC)	**2590**	378 | 2212	**2963**	1584 | 1379	npp, tree, bio10, bio17
*Leopardus wiedii **(margay)***	Near Threatened (NT)	**549**	101 | 448	**720**	393 | 327	npp, nontree, bio7, bio10
*Nasua nasua* **(coati)**	Least concern (LC)	**1978**	465 | 1513	**1906**	878 | 1028	nontree, npp, bio10, bio13
*Cerdocyon thous **(crab-eating fox)***	Least concern (LC)	**3003**	1112 | 1891	**1992**	886 | 1106	urban, tree, bio3, bio4
*Chrysocyon brachyurus***(maned wolf)**	Near Threatened (NT)	**475**	335 | 140	**386**	174 | 212	elev, grass, bio2, bio14
*Eira barbara* ***(tayra)***	Least concern (LC)	**1740**	294 | 1446	**1837**	917 | 920	npp, nontree, bio10, bio17
*Pteronura brasiliensis **(giant otter)***	Endangered (EN)	**199**	103 | 96	**21**	15 | 6	wetland, woodysavanna, bio3, bio5

**Fig. 1:**
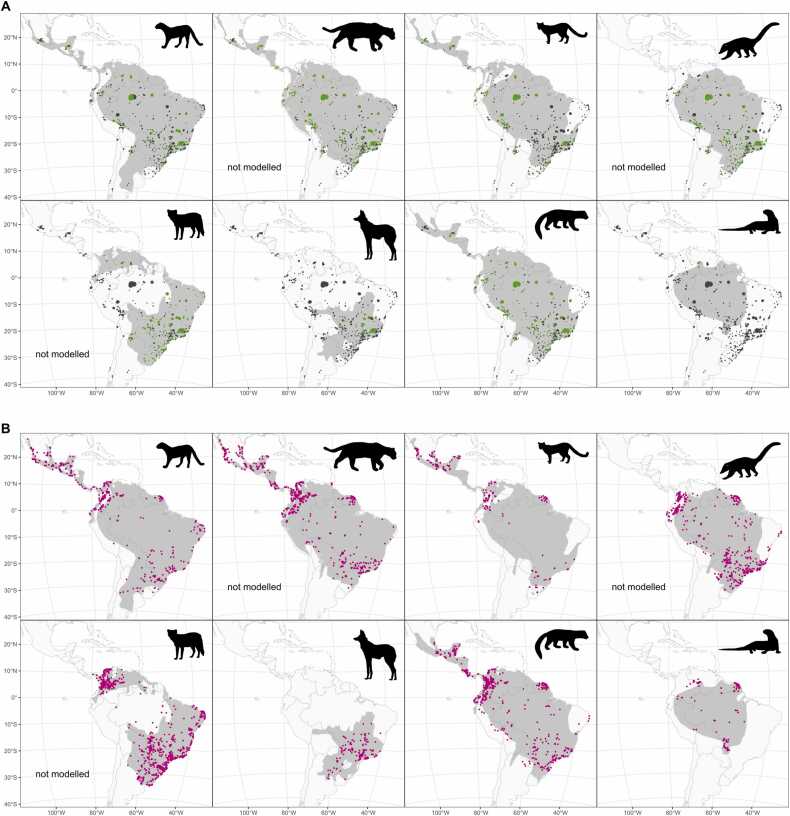
Data for the eight carnivore species used in the integrated species distribution model for the entire study period. Top: *Herpailurus yagouaroundi*, *Leopardus pardalis*, *Leopardus wiedii*, and *Nasua nasua*, and bottom: *Cerdocyon thous*, *Chrysocyon brachyurus*, *Eira barbara*, and *Pteronura brasiliensis*. (**A**) Presence-absence camera trap data from (Nagy-Reis et al., 2020) and other sources, with presences in green and absences in dark grey. (**B**) Presence-only point observations from (GBIF.org, 2023) are shown in pink. The IUCN expert map is shown in light grey for each species (IUCN, 2023). The ISDM models of *Leopardus pardalis*, *Nasua nasua*, and *Cerdocyon thous* did not converge and thus were not included in the analyses (see Results).

Presence-absence (PA) data (Table 1, Fig. 1). For all eight species, we extracted PA data from two workflows: First, we used (Nagy-Reis et al., 2020) database of neotropical carnivores records. We kept camera trap surveys (with detection and non-detection values) with geographic coordinates, information about the study sampling area, starting and ending month and year of the study, and reported the sampling effort (i.e., the number of active camera trap days). To enhance this data source, we collated and digitised 32 other camera trap survey datasets from the literature, considering the same characteristics (see Table A.2 for a complete list of sources). For each survey, we created a buffer polygon using the latitude and longitude of the survey as centroid and either the study area or the latitude/longitude precision for the studies at the sampling level of “area” as the expected area of the polygon (see the metadata in Nagy-Reis et al., 2020 for more details on these definitions). Individual polygons that overlapped were combined into one single aggregated polygon for each time period, hereafter “blob”. Finally, absences were generated for each species in those blobs where the species was not recorded. For each blob, we calculated the total surface area, the time span of the records, and the effort in camera trap days.

Presence-only (PO) data (Table 1, Fig. 1). We downloaded PO data from GBIF (GBIF.org, 2023), filtering all records with geographic coordinates and no spatial issues for 2000–2021. We further filtered these data by removing records with coordinate precision smaller than three decimal places (i.e., 0.001) and coordinate uncertainty greater than 25,000 m. To these data, we added records from (Nagy-Reis et al., 2020) that were of the type ‘Count data’ and had been surveyed using the following methods: 'Opportunistic', 'Line transect', 'Active searching', 'Roadkill', 'Museum'. Finally, we eliminated duplicates considering independent records as individuals recorded on different dates, and latitude and longitude locations. For each time period (time1 and time2), we aggregated the data to 100 ×100 km resolution grid cells (Lambert azimuthal equal-area projection; centre latitude 0º S and centre longitude 73.125º W) covering the entire Neotropics. We chose 100 km (roughly 1° cell resolution) as a compromise between computational efficiency and producing meaningful species range descriptions at a continental (macroecological) scale (Hurlbert and Jetz, 2007). To account for the uneven sampling effort between both time periods (i.e., more data are shared through GBIF over time), we calculated the ratio of the number of records between time1 and time2, using all the data available in GBIF for the eight species. We found, on average, 27 % more records in the second period. This number was used to calibrate the predictions in time2 (see section Model below).

#### 2.1.1 Expert range maps

As an additional source of information, we used expert-drawn IUCN Red List range maps (IUCN, 2023). Although our models (see below) have the flexibility to predict absences in otherwise suitable environments, they may predict (false) presences in unlikely areas for the species. In this context, range maps are an ideal source of information, as they are poor at predicting where exactly a species occurs within the range but reliably identify areas outside of the range where the species is absent. Specifically, we included the distance to the expert range maps in the model (Merow et al., 2016). Most of the IUCN range maps were generated around 2010 (2008–2016), so they do not include areas where the species could have recently colonised. To account for this, we used a value of 0 inside the range map (thus, predictions are not affected within the range) and a positive value outside the range given by the distance to the edge of the range map. This is a different approach from Merow et al. (2016), who use range maps as offsets with a fixed pre-defined coefficient, while we estimate that coefficient from the data.

#### 2.1.2 Variables describing observation effort

Thinning variables were used to explain the observation process of the presence-only records (i.e., to adjust the presence-only data for sampling effort). For each 100 ×100 km grid cell, we used data on accessibility from urban areas based on travel time (Weiss et al., 2020) and the country of origin of the record. We expected that highly accessible grid cells would have more point records than inaccessible grid cells (Hughes et al., 2021) and that differences would also vary among countries (Amano and Sutherland, 2013), as they have different data-sharing capacities and citizen-science levels of participation (Carlen et al., 2024).

#### 2.1.3 Environmental predictors

For both grid cells and blobs, we extracted the 19 bioclimatic variables, elevation (SRTM), land cover, net primary production (NPP), percentage of tree cover and percentage of non-tree vegetation. See Table A.1 for more info about each covariate’s source, resolution and time span. Land cover was processed to extract the following classes independently: urban and built-up lands, barren, water bodies, savannas, woody savannas, permanent wetlands, and grasslands. We averaged the yearly values for each covariate over the entire period and used them as a unique layer. Finally, we matched the covariates’ data to the presence-only data by averaging values within the 100 ×100 km grid cells and to the presence-absence data by averaging values within blobs.

One of the features of the Bayesian ISDM is the computational cost and, thus, the near-impossibility to do classical variable selection. To circumvent this problem, we pre-assessed the potential importance of each environmental predictor for each species using tree-based machine learning analyses (boosted trees, random forests) with the raw presence/absence for both periods combined as a response and all the environmental predictors. Finally, we performed Pearson correlations (*r*) among the top-selected variables and kept four covariates (aiming at maximum collinearity of *r*=0.6) for each species (Table 1), also taking into account the species preferences based on the available literature (Table A.3): *Herpailurus yagouaroundi* (Caso et al., 2015, de Oliveira, 1998a), *Leopardus pardalis* (de Oliveira et al., 2010, Murray and Gardner, 1997), *Leopardus wiedii* (de Oliveira, 1998b), *Nasua nasua* (Gompper and Decker, 1998), *Cerdocyon thous* (Machado and Hingst-Zaher, 2009; Tchaicka et al., 2007), *Chrysocyon brachyurus* (Dietz, 1985, Queirolo et al., 2011), *Eira barbara* (Presley, 2000), and *Pteronura brasiliensis* (Noonan et al., 2017).

### 2.2 Model

**Fig. 2:**
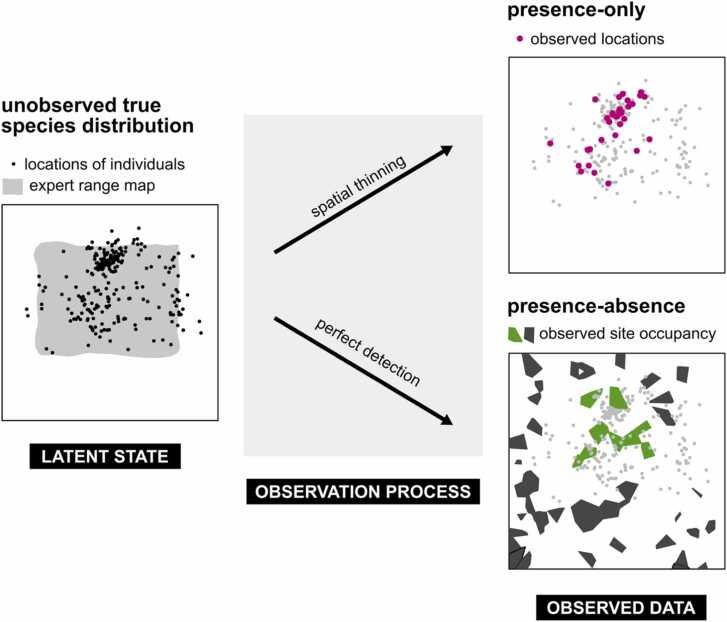
Simplified schematic of the integrated species distribution model (ISDM) used in our study. The latent state is the true (unobserved) distribution of individuals of the species. It can be modelled by a Poisson point process conditional on environmental covariates, the distance to the expert range map, time, and spatial splines (to account for missing environmental covariates and spatial autocorrelation). This true distribution is then sampled through two different observation processes, generating the presence-only and presence-absence data we observe (shown as pink dots and green and black polygons). The joint likelihood of these observed data, given the unobserved true state and uninformative priors, is then used in MCMC to calculate posterior distributions of the true state.

A full description of the model is available in Appendix B. Here is a short summary: We used an ISDM that combines three different lines of evidence (presence-only, presence-absence data, and expert range maps), accounts for sampling effort and spatial autocorrelation, and has a temporal dimension. Our model (Fig. 2) assumes that the true (unobserved) species distribution, i.e., the latent state, is an inhomogeneous Poisson point process conditional on the selected environmental covariates for each species, the distance to the expert range map, spatial splines, and time. This true distribution is then sampled through two different observation processes, generating the presence-only and presence-absence data we see (Fig. 2).

We used a similar model to Grattarola et al., (2023). Here, we introduce two novelties: (i) the distance to the edge of the expert map of the species is a covariate in the model, and (ii) we calibrate the estimated number of records per area by the overall sampling effort (measured by the ratio of the number of records between time1 and time2 for all carnivores’ data in GBIF).

#### 2.2.1 Model evaluation

We performed posterior predictive checks to evaluate the model’s fit (Conn et al., 2018) and plotted expected and observed data to compare them visually. For the PA data, we used AUC (Pearce and Ferrier, 2000) and Tjur’s R^2^ discrimination coefficient (Tjur, 2009). These values were calculated as part of each model run. For the PO data, we did residual diagnostics using the ‘DHARMa’ package (Hartig, 2022).

### 2.3 Hotspots of occupancy change

#### 2.3.1 Change of the area of occupancy

The area A of the geographic range of a species for each time period (A_time2_, A_time1_) was calculated as follows: We first integrated the point pattern intensity over each grid cell to get the expected probability of occurrence in each cell. We then summed these probabilities across all grid cells in the study area. The change in the area of occupancy over time (the number of 100 × 100 km grid-cells) was calculated as ΔA = A_time2_ - A_time1_. By “change in the area of occupancy”, we refer to the change in the total area of occupied grid cells (i.e., range size), which we distinguish from the change in (local) occupancy (i.e., probability of occurrence per grid-cell). We also calculated the uncertainty of the change (expressed as 95 % Bayesian credible intervals) and plotted it in a bivariate choropleth map together with the predicted change.

#### 2.3.2 Change in species richness

As each species’ models had a different number of iterations, first, we took 1000 samples from the posterior of the occurrence probability for each species in each grid cell and in each time period. Then, we calculated the median probability per species/grid cell, summed the individual predictions at time1 and time2 (i.e., as stacked species distribution models) and finally quantified and mapped the temporal change of species richness between periods in each grid cell.

#### 2.3.3 Beta diversity and temporal and spatial dissimilarity

We calculated (i) beta diversity as the ratio between the total diversity and the average diversity at each grid cell (Anderson et al., 2011; Whittaker, 1960), i.e., as the degree to which regional diversity exceeds local diversity, and we measure it multiplicatively, $\beta_{\mathrm{time}}=\;\gamma /{\overset{®}{\alpha }}_{\mathrm{time}}$, (ii) the spatial variation in temporal dissimilarity for each individual grid cell between the two time periods using Růžička index, and (iii) the temporal change in spatial dissimilarity as the difference in beta-diversity between par of grid cells within the same time period, using Růžička index.

### 2.4 Reproducible workflow

The data were processed in R (R Core Team, 2023). We used the ‘rnaturalearth’ package (South, 2017) to obtain Latin American countries’ spatial polygons. Spatial analyses were done using ‘sf’ (Pebesma, 2018) and ‘terra’ (Hijmans, 2022). We downloaded the MODIS data using ‘MODIStsp’ (Busetto and Ranghetti, 2016). The ISDM was run using ‘R2jags’ (Su and Yajima, 2020), and the maps were prepared with ‘tmap’ (Tennekes, 2018). Beta diversity was calculated using *vegdist* in ‘vegan’ package (Oksanen et al., 2013). The workflow for each species was split into five Quarto notebooks, including 1) data generation, 2) covariates’ selection, 3) data preparation for modelling, 4) model run, and 4) model outputs. All this is accessible in Grattarola (2024) or at our GitHub repository https://github.com/bienflorencia/hotspots-of-change.

## 3 Results

We fitted a separate ISDM for each mammal species and revealed their geographic range dynamics in the Neotropics over the last two decades (Figure A.1). Good convergence was reached for all model parameters (Rhat <1.1). Of the eight species, five were well supported based on model performance (i.e., AUC, Tjur R^2^, and residual diagnostics fit). We were not able to assess the distribution range of *Leopardus pardalis*, *Cerdocyon thous*, and *Nasua nasua* because the PO residuals indicated poor fit, suggesting that the observed data did not align well with the fitted model (Figure A.2). Thus, we excluded this species from the occupancy change analyses. Average Tjur's R^2^ was 0.289, and AUC was 0.708 for the PA data (Table A.4), and we saw an overall reasonable fit for the PO data (Figure A.2).

### 3.1 Changes in occupancy and the area of occupancy of each species

**Fig. 3:**
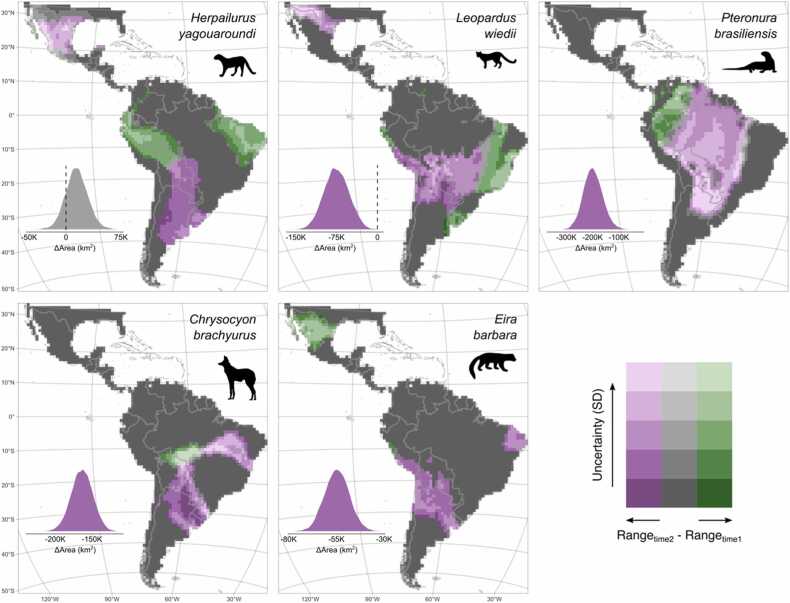
Mapped changes in the occupancy (probability of occurrence in a grid cell) and area of occupancy (range size) of species. The change between the two time periods (2000–2013 and 2014–2021) is split by the uncertainty of the prediction; darker pink and darker green colours show highly certain losses and gains, respectively. The distribution of the area of change is shown in the lower left corner for each species.

The changes in the area of occupancy (the occupied area of 100 × 100 km grid-cells) varied between species, ranging from −2,000,000 km^2^ to 146,000 km^2^. They were predominantly negative (Fig. 3), meaning that most species (except for *Herpailurus yagouaroundi*) decreased their probability of occurrence relative to the initial period.

We found that the jaguarundi (*Herpailurus yagouaroundi*) has contracted its southern range limits in Argentina and south Brazil while maintaining its presence in central Brazil and the north of South America and expanding its range in the northeast of Brazil (between Cerrado and Caatinga biomes) and the western Amazon (Fig. 3). We saw a non-significant increase in the species range between the two periods, with a median change in the area of occupancy of 146,000 km^2^ (14.6 grid-cells of 100x100km; CI = −22.4, 54.2). The margay (*Leopardus wiedii*) showed range decreases in south Peru and the Chaco and Pantanal regions (Bolivia, Paraguay, north of Argentina and south-western Brazil), and range expansions in the Uruguayan savannah (Uruguay and its borders with Argentina and Brazil), part of Cerrado and Caatinga regions, the north of the Atlantic Forest (Brazil), and the north of Peru and Ecuador. Between both periods, the species contracted its range in −756,000 km^2^ (75.6 grid-cells; CI = −129, −21.1). For the maned wolf (*Chrysocyon brachyurus*), we saw large geographic range contractions that concentrated in the Chaco and Uruguayan savannah regions (Uruguay, north of Argentina, and south Paraguay) and weak expansions over the south-west Amazon moist forests (north Bolivia and south-west Brazil). The median change in the area of occupancy was 1,640,000 km^2^ (164 grid-cells; CI = −200, −129). We found that the tayra (*Eira barbara*) has contracted on its southern range limit (central Argentina) and expanded on its northern limit (Mexico). The median decrease in the area of distribution for the tayra was −548,000 km^2^ (54.8 grid-cells; CI = −70.7, −39.4). The giant otter (*Pteronura brasiliensis*) was the species with the largest range loss. Contractions were widespread along the species distribution (mainly in the Amazon basin), with few areas of unchanged areas concentrated in Guiana lowland moist forests and range expansions in the western limits of the species range. The giant otter shrunk its range by a median of 2,000,000 km^2^ (200 grid-cells; CI = −283, −106).

### 3.2 Change in species richness

**Fig. 4:**
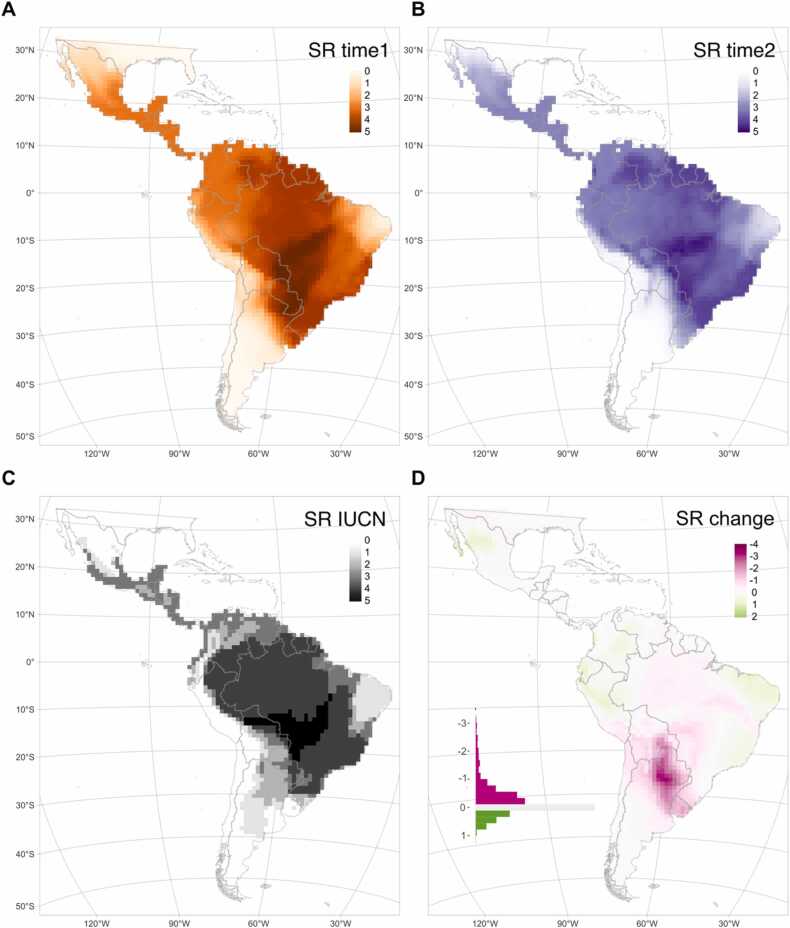
Patterns of species richness (SR) and SR change. Including maned wolf (*Chrysocyon brachyurus*), giant otter (*Pteronura brasiliensis*), jaguarundi (*Herpailurus yagouaroundi*), tayra (*Eira barbara*), and margay (*Leopardus wiedii*). (**A**) Species richness in time1 (2000–2013), (**B**) species richness in time2 (2014–2021), (**C**) species richness according to the IUCN expert range maps (IUCN, 2023), and (**D**) change in species richness between both time periods (pink regions indicate species losses and green regions indicate species gains).

Species richness at each time period (Fig. 4a,b), calculated as the average richness across each period per grid cell, showed an overall similar pattern to that expected by IUCN range maps (Fig. 4c). Diversity of the five species peaked between −10 and −25 degrees south and −55 and −35 degrees west and declined towards the west of South America, northeast of Brazil and the north of Mexico. The temporal change in species richness was unevenly distributed across the continent (Fig. 4c). Losses were accumulated in a region covering Uruguay, the north of Argentina, Paraguay and south Bolivia, and were mostly driven by the contraction of the ranges of *Chrysocyon brachyurus*, *Herpailurus yagouaroundi*, *Eira barbara*, and *Leopardus wiedii* (see are of occupancy changes in Fig. 3). Gains were less conspicuous and more geographically dispersed, with notable centres in the Caatinga and the Atlantic Forest regions (northeast and southwest of Brazil), the tropical Andes (central and north Peru, west Ecuador and Colombia) and north-west Mexico (Fig. 4c).

### 3.3 Beta diversity and spatial and temporal dissimilarity

**Fig. 5:**
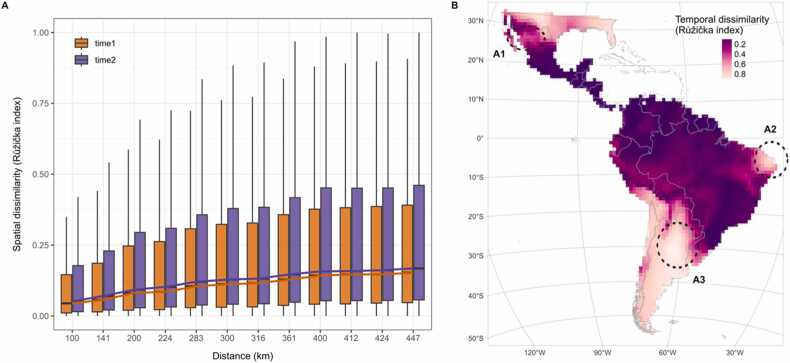
Change in temporal dissimilarity and spatial dissimilarity of five carnivore species. Measured with Růžička index, between 2000 and 2013 and 2014–2021. (**A**) Temporal change of spatial dissimilarity, i.e., between each time period at the same grid cell. Dissimilarity is higher in time2 and increases with distance for both periods; time1 is shown in orange, and time2 in purple. Lines connecting the median values are also shown. (**B**) Spatial variation in temporal dissimilarity, i.e., between pairs of grid cells within the same time period. High dissimilarity between time1 and time2 is represented in light pink and low in dark purple. A1, A2 and A3 highlight areas of interest due to high dissimilarity and richness (within or on the boundaries of accumulated species ranges).

Beta diversity, the ratio between the total diversity and the average diversity at each grid cell, increased from $\beta_{\mathrm{time}1}$=1.911 (±3.321) to $\beta_{\mathrm{time}2}$=2.088 (±3.408). We also saw an increase in temporal change of spatial dissimilarity with distance between periods, with time2 being higher than time1 (Fig. 5a). Temporal dissimilarity of species composition between time1 and time2, measured by the Růžička index, concentrated around locations with a high concentration of range boundaries (Fig. 5b), particularly in the northwest of Mexico (Fig. 5b, A1), northeast Brazil (A2), and the northeast of Argentina (A3). The peaks of temporal dissimilarity in Mexico (A1) and Brazil (A2) are also areas of change in species composition. In contrast, the peak in the north of Argentina overlapped the hotspot of species richness loss (Fig. 4c). A closer look at the first case (A1) reveals a gain of *Eira barbara* and a loss of *Herpailurus yagouaroundi*, while the second case (A2) is explained by the gain of *Herpailurus yagouaroundi* and the loss of *Eira barbara*.

## 4 Discussion

There is a high demand for empirical assessments of how nature has been changing in response to anthropogenic pressures. Yet even the most high-profile reports (IPBES, 2019) rely either on indirect evidence (e.g. habitats degrade and thus biodiversity must decline), on projection scenarios (e.g. this is how climate changes and biodiversity will follow), or on reports from small (local) spatial grains (Blowes et al., 2019). In contrast to these, our study provides the first *direct* continent-wide, multi-species and continuous map of hotspots of temporal change in the Neotropics over the last two decades. By focusing on the five carnivores’ entire distribution, we identified variations in species’ occupancy areas, species richness, and species composition. Most species, one of them listed as endangered and two near threatened (Table 1), underwent range contractions in the last twenty years, their diversity decreased over time, and species composition underwent spatial differentiation (sensu Blowes et al., 2022, i.e., dissimilarity among assemblages increased). The type of changes and directions differed among regions and countries, and we suggest that this variation can be linked to the ongoing land use changes in the Neotropical region (Jaureguiberry et al., 2022). Global targets, such as the Kunming-Montreal Global Biodiversity Framework, demand up-to-date biodiversity knowledge to be used for urgent conservation action. Our study provides evidence that shows where and how prominent the declines are in different parts of the continent. Thus, our analysis can contribute to National Biodiversity Assessments and help prioritise areas for immediate conservation action that can be tailored to each species.

We found the most important changes in three specific areas: west of Mexico (Sierra Madre Occidental and Pacific Lowlands) and northeast of Brazil (Caatinga), with high temporal dissimilarity, and the north of Argentina (Pampa and Chaco/Espinal), with high temporal dissimilarity and also species loss. The west of Mexico, partially located in the Mexican transition zone where the Neotropical and the Nearctic regions overlap (Morrone, 2017), has not been the part of the country most affected by land use change (González-Abraham et al., 2015). However, it has one of the lowest densities of protected areas (Koleff et al., 2009), with identified gaps specifically for carnivores’ conservation (Gonzalez and Harris, 2024, Valenzuela Galván and Vázquez, 2008). In particular, the tropical and sub-tropical dry forests of this region, some of the most diverse in the Americas (Sosa et al., 2018), have been disregarded in terms of conservation policies in comparison to tropical evergreen forests in the country (Mendoza-Ponce et al., 2019), and identified as vulnerable under projected climatic scenarios (Ureta et al., 2022). This shortfall of conservation policies could explain the pattern we observe.

The Caatinga is the largest seasonal tropical dry forest in South America (Da Silva et al., 2017). Although the vegetation in this region is adapted to extreme temperature conditions, it is expected to be highly affected by climate change (Moura et al., 2023a; Silva et al., 2019) as more than 90 % of the Caatinga is under moderate to high susceptibility to desertification (Vieira et al., 2021). Mammals here also need to adapt to unpredictable water availability while navigating a landscape heavily impacted by human activity (Dias et al., 2019, Fox-Rosales and de Oliveira, 2022). Almost half of Caatinga has been converted to agriculture and ranching, with most remaining areas fragmented and disturbed, and only 7.5 % under protection, largely in small, private reserves (Antongiovanni et al., 2020; Baumann et al., 2017).

Like the Caatinga, the Chaco/Espinal and Pampa regions are not among the biodiversity hotspots of the Neotropics (Myers et al., 2000); they represent areas of medium species richness values. Because of this and because they are largely less-forested ecosystems (i.e., mainly a mosaic of grasslands, shrublands and woodlands), they have been historically neglected (Grau et al., 2015; Vieira et al., 2019). Importantly, these southern lowland regions have experienced severe land use changes over the last three decades (Piquer-Rodríguez et al., 2018, Stanimirova et al., 2022). The Chaco has lost 14.5 % of its natural vegetation (1,440,000 km^2^) compared to 1985, with the greatest loss located in Paraguay (Proyecto Mapbiomas Chaco, 2023), while the Pampa has lost 11.8 % (700,000 km^2^), mainly of native grasslands (Baeza et al., 2022, Proyecto MapBiomas, 2022). The conspicuous species loss in these areas could be a consequence of such profound land use changes (Romero-Muñoz et al., 2021).

We found diverse types of change in each individual species. The *Herpailurus yagouaroundi* was the only species that did not experience net declines in its area of occupancy. The disparity between our new findings and previous results, suggesting a slight increase (Grattarola et al., 2023), can be attributed to the incorporation of the species expert range map in our current model. Including this expert-derived information may constrain the predictions, leading to a more accurate representation of the species' actual occupancy dynamics. The increase in the area of occupancy of *H. yagouaroundi* towards the Caatinga region on the border with the Cerrado can be explained by the strong wet/dry climate there, which the jaguarundi prefers (Espinosa et al., 2018). Likewise, the recent reports of the species there (Fox-Rosales and de Oliveira, 2022), can also reflect its flexibility and tolerance to human settlements. This expansion pattern aligns with Moura et al. (2023b), who projected an increase in habitat suitability for the species by 2060 there. However, we saw a sharp contraction in the southern limit of its distribution range, which aligns with the drastic land use changes occurring in this area (Baeza et al., 2022; Piquer-Rodríguez et al., 2018) and the scarce presence of the species in the southernmost distribution limit (Luengos Vidal et al., 2017). Thus, the recent first recordings of the species in Uruguay (Grattarola et al., 2016) could either be an erratic detection of the species or a lack of past sampling effort in the area, but not the expansion of the species. Overall, this is one of the most broadly distributed carnivores in the Neotropics, and yet its distribution dynamics remains understudied (Gálvez et al., 2023).

In the Uruguayan savanna region (Uruguay, south Brazil and Argentina on the border with Uruguay), we observed an opposite trend for the *Leopardus wiedii*, whose occupancy increased over time. This aligns with previous studies that have seen an extension in the distribution of this species in Uruguay over time (Schiaffini et al., 2023). Categorised as Near Threatened, *L. wiedii* is highly dependent on trees, and the few forested areas of these grasslands in the region may be key for the species’ conservation planning (Espinosa et al., 2018). The species also expanded over the Brazilian Atlantic dry forest on the border with Caatinga, which is moderately predicted by habitat suitability analyses (Espinosa et al., 2018) and supported by recent detections of the species there (Marinho et al., 2024, Meira et al., 2018). The main reductions in the area of occupancy for *L. wiedii* were in the western part of the Chaco, an heterogeneous area characterised as dry open woodland. There are no abundance or occupancy studies reported for the species in this region (Gálvez et al., 2023); however, the high levels of deforestation due to agricultural expansion may be negatively impacting the species populations there (Fehlenberg et al., 2017, Mosciaro et al., 2023).

*Chrysocyon brachyurus* is the largest South American canid and a near-threatened species (Paula and De Matteo, 2015). *C. brachyurus* showed a stable occupancy in the Cerrado, yet large declines towards the south of its range, a continuation of a process that had already been documented prior to the year 2000 (Queirolo et al., 2011). Recent studies, however, have opportunistically reported the species at the southern edge of its range in areas with significant land transformations and human-wildlife conflict (Kasper et al., 2023, Orozco et al., 2023). Authors suggest that these individuals may be transient rather than establishing permanent populations, emphasising the need to focus conservation efforts there (Kasper et al., 2023). *C. brachyurus*, however, has expanded its north-western distribution into the forests of Amazonia. This could be explained by the conversions of broad areas of the lower Amazon to livestock pastures (Souza et al., 2020), giving the species larger open areas to occupy.

The *Eira barbara*’s area of occupancy also declined, in particular around the species’ southern limit and towards the Caatinga in northeast Brazil, aligning with the projected range shifts of Moura et al. (2023b). *E. barbara*’s area of occupancy in the centre of Mexico showed an increase, although the species is uncommon there and considered endangered in the whole country (Norma Oficial Mexicana, 2010). The recent range expansions documented in south and central Mexico could, however, support our findings (García et al., 2016, Ruiz-Gutiérrez et al., 2017). Yet arid environments, such as those in the Chihuahua desert of Mexico, have been recognised as low-suitability habitats for *E. barbara* (Schiaffini et al., 2017).

Finally, *Pteronura brasiliensis*, one of the most endangered mammals of the Neotropics (Noonan et al., 2017), was the species with the most prominent declines in the area of occupancy, with few areas of expansion that were located in the upper Amazon. There is evidence that *P. brasiliensis* may be recovering in this area, around north Perú and northeastern Ecuadorian Amazon (Groenendijk et al., 2014), but there are also reports of population declines in western Colombia and south Perú and within the rest of the entire range (Groenendijk et al., 2022). Critically, the species is considered locally extinct throughout most of its historical eastern and southern range (Garbino et al., 2022) and most populations of *P. brasiliensis* in the Orinoco, Amazon, and Parana basins are fragmented and isolated (Noonan et al., 2017). Despite slowly recovering from decades of hunting for the pelt trade, deforestation of the Amazon and contamination of water bodies (e.g., by mining) are, in any case, making the species more vulnerable (Brum et al., 2021, Cianfrani et al., 2018).

The presence-absence data we used are more evenly spread than presence-only data, and both data types are spatially complementary. Therefore, they jointly present low imbalances in the geographic space they cover. However, a question may arise whether the estimated occupancy change is real and not a mere reflection of survey effort. Here are the reasons why the latter is unlikely: (1) our predicted ranges align with the current expert knowledge (IUCN range) and not with the perceived imbalance in the raw data, (2) we account for several facets of the effort in the model, and (3) since the model is Bayesian, an area with insufficient data translates in high prediction uncertainty, which we then report.

We show that the model of *Herpailurus yagouaroundi* originally developed by Grattarola et al., (2023) can incorporate expert range maps and be applied to four other carnivore species; however, we were not able to fit it for three species, *Leopardus pardalis*, *Cerdocyon thous*, and *Nasua nasua*, because the model showed poor residual diagnostics fit (Figure A.2). This may be because they are widespread habitat generalists that do not respond to our broad-scale environmental covariates or exhibit a clear spatially structured trend. Still, classical model performance metrics such as AUC and R² are difficult to interpret in hierarchical models that incorporate both observation and process sub-models (including ISDMs and occupancy models as described by MacKenzie et al., 2018). These metrics should not be applied in the same way as in classical SDMs. The challenge arises because the model estimates the unobserved true occupancy, which represents the actual occupancy, assuming the model is correct. Consequently, the only valid dataset for calculating AUC and R² would need to accurately reflect this unobserved true occupancy, demanding data from sites where every individual is detected and identified. Such comprehensive data are practically unattainable. Even though the analysis of trends in the remaining five species may seem limited, it still represents the first example of how temporal changes of occupancy and diversity can be scaled up to entire ranges and multiple species with limited and heterogeneous data. In this study we provide a new approach to evaluate past species range dynamics based on multiple lines of evidence and using ISDMs, which can be employed over more species in the future, particularly in under-sampled regions.

In all, we put a temporal perspective on the continental-wide distributions of carnivore species in the Neotropics and discussed potential drivers of change. We unveiled the species’ large-scale range dynamics, a key step to implementing conservation measures at the local scale. Likewise, our study highlights the need for global conservation efforts by providing a critical understanding of how changes in the Neotropics are reshaping biodiversity, offering insights that are essential for mitigating the worldwide impacts of ecosystem degradation. With this temporal multi-species approach, we have paved the way to a dynamic macroecology which no longer produces static range polygons or maps from species distribution models. Instead, we envision a scenario where field guides, information signs in zoological gardens and ecotourism areas, come with both contemporary and historical distributions. This is necessary in order to grasp the full extent of the ongoing global biodiversity change, particularly for the general public.

## Ethics Statement

This manuscript does not include human or animal research.

## Data Accessibility Statement

The data used for this study are openly available at Nagy-Reis et al., (2020) (https://doi.org/10.1002/ecy.3128) and GBIF.org (2023) (https://doi.org/10.15468/DL.TVVZDQ). A list of the additional sources gathered for this study can be seen in Table A.2. To see the code for all the analyses and the workflow followed for each species, including data preparation, covariates selection, model run and model outputs, access Grattarola (2024) or our GitHub repository https://github.com/bienflorencia/hotspots-of-change.

## Funding

10.13039/501100000780European Union (ERC, BEAST, 101044740).

## Declaration of Competing Interest

The authors declare that they have no known competing financial interests or personal relationships that could have appeared to influence the work reported in this paper.
