# Supplementary material for: A continental-wide decline of occupancy and diversity in five Neotropical carnivores
Source: Glob Ecol Conserv. 2024 Nov;55:e03226. doi: 10.1016/j.gecco.2024.e03226 (PMC11513410; doi:10.1016/j.gecco.2024.e03226)
Supplement: Supplementary file 1 — Appendix A Supplementary material [file mmc1.docx]

Appendix A

Contents

Table A.1 – Environmental predictors' definition, source, resolution, and temporal span.

Table A.2 – List of presence-absence data sources gathered to complement Nagy-Reis et al. (2020) database.

Table A.3 – List of species preferences based on the available literature and selected covariates for the ISDMs.

Table A.4 – Model evaluation for the presence-absence data measured by Tjur's R2 and AUC.

Figure A.1 – Predicted occupancy probability of the species at the two time periods (2000 to 2013 and 2014 to 2021).

Figure A.2 – Model evaluation for the presence-only data assessed.

References

**Table A.1.** Environmental predictors' definition, source, resolution, and temporal span.

| **Variable** | **Definition** | **Source** | **Spatial resolution** | **Temporal span** |
| --- | --- | --- | --- | --- |
| bio1 | Annual Mean Temperature | WorldClim V2.1 (Fick & Hijmans 2017) | 30s | 1970-2000 |
| bio2 | Mean Diurnal Range (Mean of monthly (max temp - min temp)) | WorldClim V2.1 (Fick & Hijmans 2017) | 30s | 1970-2000 |
| bio3 | Isothermality (BIO2/BIO7) (×100) | WorldClim V2.1 (Fick & Hijmans 2017) | 30s | 1970-2000 |
| bio4 | Temperature Seasonality (standard deviation ×100) | WorldClim V2.1 (Fick & Hijmans 2017) | 30s | 1970-2000 |
| bio5 | Max Temperature of Warmest Month | WorldClim V2.1 (Fick & Hijmans 2017) | 30s | 1970-2000 |
| bio6 | Min Temperature of Coldest Month | WorldClim V2.1 (Fick & Hijmans 2017) | 30s | 1970-2000 |
| bio7 | Temperature Annual Range (BIO5-BIO6) | WorldClim V2.1 (Fick & Hijmans 2017) | 30s | 1970-2000 |
| bio8 | Mean Temperature of Wettest Quarter | WorldClim V2.1 (Fick & Hijmans 2017) | 30s | 1970-2000 |
| bio9 | Mean Temperature of Driest Quarter | WorldClim V2.1 (Fick & Hijmans 2017) | 30s | 1970-2000 |
| bio10 | Mean Temperature of Warmest Quarter | WorldClim V2.1 (Fick & Hijmans 2017) | 30s | 1970-2000 |
| bio11 | Mean Temperature of Coldest Quarter | WorldClim V2.1 (Fick & Hijmans 2017) | 30s | 1970-2000 |
| bio12 | Annual Precipitation | WorldClim V2.1 (Fick & Hijmans 2017) | 30s | 1970-2000 |
| bio13 | Precipitation of Wettest Month | WorldClim V2.1 (Fick & Hijmans 2017) | 30s | 1970-2000 |
| bio14 | Precipitation of Wettest Month | WorldClim V2.1 (Fick & Hijmans 2017) | 30s | 1970-2000 |
| bio15 | Precipitation Seasonality (Coefficient of Variation) | WorldClim V2.1 (Fick & Hijmans 2017) | 30s | 1970-2000 |
| bio16 | Precipitation of Wettest Quarter | WorldClim V2.1 (Fick & Hijmans 2017) | 30s | 1970-2000 |
| bio17 | Precipitation of Driest Quarter | WorldClim V2.1 (Fick & Hijmans 2017) | 30s | 1970-2000 |
| bio18 | Precipitation of Warmest Quarter | WorldClim V2.1 (Fick & Hijmans 2017) | 30s | 1970-2000 |
| bio19 | Precipitation of Coldest Quarter | WorldClim V2.1 (Fick & Hijmans 2017) | 30s | 1970-2000 |
| elevation | Elevation (SRTM) | WorldClim V2. (Fick & Hijmans 2017) | 1km |  |
| land cover | Land cover classes | NASA MODIS Terra (MCD12Q1) (Friedl & Sulla-Menashe 2019) | 500m | 2000 to 2020 |
| npp | Net primary production | NASA MODIS Terra (M*D17A3HGF) (Running & Zhao 2019) | 500m | 2000 to 2020 |
| tree | Percentage of Tree Cove | NASA MODIS Terra (MOD44B) (DiMiceli et al. 2015) | 250m | 2000 to 2020 |
| nontree | Percentage of No Tree Cover | NASA MODIS Terra (MOD44B) (DiMiceli et al. 2015) | 250m | 2000 to 2020 |
| nonveg | Percentage of Non Tree Vegetation Cover | NASA MODIS Terra (MOD44B) (DiMiceli et al. 2015) | 250m | 2000 to 2020 |

Table A.2. List of presence-absence data sources gathered to complement Nagy-Reis et al. (2020) database.

| Year | Author | Title | Item Type | Publication Title | DOI/URL |
| --- | --- | --- | --- | --- | --- |
| 2020 | Jansen, Martin; Engler, Marc; Blumer, Luka Moritz; Rumiz, Damián I.; Aramayo, José Luis; Krone, Oliver | A camera trapping survey of mammals in the mixed landscape of Bolivia’s Chiquitano region with a special focus on the Jaguar | journalArticle | Check List | 10.15560/16.2.323 |
| 2020 | Teal, Randy Charles | A camera trapping study on the spatial distribution of terrestrial mammals within a human-dominated landscape in the Pacific lowlands of Costa Rica | thesis |  | 10.25365/thesis.62291 |
| 2019 | Huguenin, Jade; Ferreira, Juliana Rodrigues; Moreira, Danielle Oliveira; Gatti, Andressa; Mendes, Sérgio Lucena; Zanin, Marina | An updated species list of medium and large-sized mammals of The Augusto Ruschi Biological Reserve, using a novel sampling design | journalArticle | Oecologia Australis | 10.4257/oeco.2019.2301.06 |
| 2016 | Paolino, Roberta Montanheiro; Versiani, Natalia Fraguas; Pasqualotto, Nielson; Rodrigues, Thiago Ferreira; Krepschi, Victor Gasperotto; Chiarello, Adriano Garcia | Buffer zone use by mammals in a Cerrado protected area | journalArticle | Biota Neotropica | 10.1590/1676-0611-BN-2014-0117 |
| 2020 | Huarcaya, Ruthmery Pillco; Beirne, Christopher; Rojas, Shirley Jennifer Serrano; Whitworth, Andrew | Camera trapping reveals a diverse and unique high-elevation mammal community under threat | journalArticle | Oryx | 10.1017/S0030605318001096 |
| 2018 | Porfirio, Grasiela; Foster, Vania; Sarmento, Pedro; Fonseca, Carlos | Camera traps as a tool for Carnivore conservation in a mosaic of Protected Areas in the Pantanal wetlands, Brazil | journalArticle | Nature Conservation Research | 10.24189/ncr.2018.035 |
| 2020 | Botts, Ryan T; Eppert, Amy A; Wiegman, Timothy J; Rodriguez, Abner; Blankenship, Steven R; Asselin, Ellen M; Garley, Wyatt M; Wagner, Abigail P; Ullrich, Sierra E; Allen, Gabrielle R; Mooring, Michael S | Circadian activity patterns of mammalian predators and prey in Costa Rica | journalArticle | Journal of Mammalogy | 10.1093/jmammal/gyaa103 |
| 2021 | Morales-Martínez, Darwin M.; Atuesta-Dimian, Natalia; Martínez-Medina, Daniela; Gutiérrez-Sanabria, Diego R.; Rodríguez-Posada, Miguel E. | Completeness of rapid assessments of medium and large mammal diversity in the northwestern Amazon in Colombia | journalArticle | Acta Amazonica | 10.1590/1809-4392202100741 |
| 2018 | González-Gallina, Alberto; Hidalgo-Mihart, Mircea G.; Castelazo-Calva, Víctor | Conservation implications for jaguars and other neotropical mammals using highway underpasses | journalArticle | PLOS ONE | 10.1371/journal.pone.0206614 |
| 2018 | Pardo Vargas, Lain Efrén | Diversity and habitat use of medium-large sized mammals across oil palm landscapes in the Llanos region of Colombia | thesis |  | 10.25903/5be8adb35232e |
| 2018 | Marques, Rosane Vera; Fabián, Marta Elena | Diversity of medium and large neotropical mammals in an area of mixed rain forest | journalArticle | Acta Scientiarum. Biological Sciences | 10.4025/actascibiolsci.v40i1.40910 |
| 2008 | Srbek-Araujo, A. C.; Chiarello, A. G. | Domestic dogs in Atlantic forest preserves of south-eastern Brazil: a camera-trapping study on patterns of entrance and site occupancy rates | journalArticle | Brazilian Journal of Biology | 10.1590/S1519-69842008000400011 |
| 2021 | Mena, José Luis; Yagui, Hiromi; Tejeda, Vania; Bonifaz, Emilio; Bellemain, Eva; Valentini, Alice; Tobler, Mathias W.; Sánchez-Vendizú, Pamela; Lyet, Arnaud | Environmental DNA metabarcoding as a useful tool for evaluating terrestrial mammal diversity in tropical forests | journalArticle | Ecological Applications | 10.1002/eap.2335 |
| 2022 | Vargas Soto, Juan S.; Beirne, Christopher; Whitworth, Andrew; Cruz Diaz, Juan Carlos; Flatt, Eleanor; Pillco-Huarcaya, Ruthmery; Olson, Erik R.; Azofeifa, Alejandro; Saborío-R, Guido; Salom-Pérez, Roberto; Espinoza-Muñoz, Deiver; Hay, Leslie; Whittaker, Lawrence; Roldán, Carmen; Bedoya-Arrieta, Ricardo; Broadbent, Eben North; Molnár, Péter K. | Human disturbance and shifts in vertebrate community composition in a biodiversity hotspot | journalArticle | Conservation Biology | 10.1111/cobi.13813 |
| 2019 | Antunes, Ana Carolina; Baccaro, Fabrício; Caetano Andrade, Victor Lery; Ramos, José Ferreira; Da Silva Moreira, Roberto; Barnett, Adrian A | Igapó seed patches: a potentially key resource for terrestrial vertebrates in a seasonally flooded forest of central Amazonia | journalArticle | Biological Journal of the Linnean Society | 10.1093/biolinnean/blz101 |
| 2021 | Hernandez-Hernandez, Julio C.; Chavez, Cuauhtémoc | Inventory of medium-sized and large mammals in La Encrucijada Biosphere Reserve and Puerto Arista Estuarine System, Chiapas, Mexico | journalArticle | Check List | 10.15560/17.4.1155 |
| 2020 | Pierre, Meshach A.; Ignacio, Leroy; Paemelaere, Evi A. D. | Large- and medium-bodied terrestrial mammals of the Upper Berbice region of Guyana | journalArticle | Check List | 10.15560/16.5.1229 |
| 2021 | Arévalo-Sandi, Alexander Roldán; Gonçalves, André Luis Sousa; Onizawa, Kota; Yabe, Tsuneaki; Spironello, Wilson Roberto | Mammal diversity among vertical strata and the evaluation of a survey technique in a central Amazonian forest | journalArticle | Papéis Avulsos de Zoologia | 10.11606/1807-0205/2021.61.33 |
| 2021 | Briseño-Hernández, Iván; Naranjo, Eduardo J. | Outstanding records of mammals from two protected areas of central Guerrero, México | journalArticle | Therya Notes | 10.12933/therya_notes-21-43 |
| 2022 | Assis, William Oliveira de; Santos, Filipe Martins; Nascimento, Leonardo França do; Barreto, Wanessa Teixeira Gomes; Nantes, Wesley Arruda Gimenes; Fonseca, Carlos; Herrera, Heitor Miraglia; Porfirio, Grasiela Edith de Oliveira | Medium- and large-sized mammals at The Urucum Massif in the Brazilian Pantanal: Camera trap as an effective sampling method to estimate species richness, relative abundance, and activity patterns | journalArticle | Oecologia Australis | 10.4257/oeco.2022.2601.03 |
| 2018 | Hoskins, Hannah M. J.; Burdekin, Oliver J.; Dicks, Kara; Slater, Kathy Y.; McCann, Niall P.; Jocque, Merlijn; Castañeda, Franklin; Reid, Neil | Non-volant mammal inventory of Cusuco National Park, north-west Honduras: reporting the presence of Jaguar, Panthera onca (Linnaeus, 1758), and demonstrating the effects of zonal protection on mammalian abundance | journalArticle | Check List | 10.15560/14.5.877 |
| 2017 | Mendes-Oliveira, Ana Cristina; Peres, Carlos A.; Maués, Paula Cristina R. de A.; Oliveira, Geovana Linhares; Mineiro, Ivo G. B.; Maria, Susanne L. Silva de; Lima, Renata C. S. | Oil palm monoculture induces drastic erosion of an Amazonian forest mammal fauna | journalArticle | PLOS ONE | 10.1371/journal.pone.0187650 |
| 2018 | Alvarenga, Guilherme Costa; Ramalho, Emiliano Esterci; Baccaro, Fabrício Beggiato; Rocha, Daniel Gomes da; Ferreira-Ferreira, Jefferson; Bobrowiec, Paulo Estefano Dineli | Spatial patterns of medium and large size mammal assemblages in várzea and terra firme forests, Central Amazonia, Brazil | journalArticle | PLOS ONE | 10.1371/journal.pone.0198120 |
| 2019 | Castillo-Figueroa, Dennis; Martínez-Medina, Daniela; Rodríguez-Posada, Miguel E.; Bernal-Vergara, Sandra | Structural differences in mammal assemblages between savanna ecosystems of the Colombian Llanos | journalArticle | Papéis Avulsos de Zoologia | 10.11606/1807-0205/2019.59.14 |
| 2020 | Beal, Maxwell R. W.; Matzinger, Parker J.; Saborío-R, Guido; Bristan, Jonathan Noguera; Olson, Erik R. | Survey of medium-sized and large mammals of Piedras Blancas National Park, Costa Rica | journalArticle | Check List | 10.15560/16.4.939 |
| 2017 | Luna, Rodolfo Burgos de; Reyes, Andrés Felipe Alfonso; Lucena, Leandro Ricardo Rodrigues de; Pontes, Antonio Rossano Mendes | Terrestrial mammal assemblages in protected and human impacted areas in Northern Brazilian Amazonia | journalArticle | Nature Conservation | 10.3897/natureconservation.22.17370 |
| 2016 | Paschoal, Ana Maria O.; Massara, Rodrigo L.; Bailey, Larissa L.; Kendall, William L.; Doherty Jr., Paul F.; Hirsch, André; Chiarello, Adriano G.; Paglia, Adriano P. | Use of Atlantic Forest protected areas by free-ranging dogs: estimating abundance and persistence of use | journalArticle | Ecosphere | 10.1002/ecs2.1480 |
| 2020 | Akkawi, Paula; Villar, Nacho; Mendes, Calebe P; Galetti, Mauro | Dominance hierarchy on palm resource partitioning among Neotropical frugivorous mammals | journalArticle | Journal of Mammalogy | 10.1093/jmammal/gyaa052 |
| 2015 | Michalski, Lincoln José; Norris, Darren; Oliveira, Tadeu Gomes de; Michalski, Fernanda | Ecological Relationships of Meso-Scale Distribution in 25 Neotropical Vertebrate Species | journalArticle | PLOS ONE | 10.1371/journal.pone.0126114 |
| 2021 | Silva, D. S.; Ribeiro, M. V.; Soares, F. H. | Medium and large-sized mammals of a private protected wetland in the Cerrado-Amazon biological corridor, Brazil | journalArticle | Brazilian Journal of Biology | 10.1590/1519-6984.243666 |
| 2013 | Carvalho, W. D.; Adania, C. H.; Esbérard, C. E. L. | Comparison of two mammalian surveys made with camera traps in southeastern Brazil, focusing the abundance of wild mammals and domestic dogs | journalArticle | Brazilian Journal of Biology | 10.1590/S1519-69842013000100005 |
| 2012 | Blake, John G; Mosquera, Diego; Loiselle, Bette A; Swing, Kelly; Guerra, Jaime; Romo, David | Temporal activity patterns of terrestrial mammals in lowland rainforest of Eastern Ecuador | journalArticle | Ecotropica | http://www.soctropecol.eu/PDF/Ecotropica_2012/Blake_et_al_2012_Ecotropica_18_2.pdf |

**Table A.3**. List of species preferences based on the available literature, and selected covariates for the ISDMs.

| **Species** | **Species’ preferences** | **References** | **Selected covariates** |
| --- | --- | --- | --- |
| *Herpailurus yagouaroundi* (**jaguarundi**) | associated with forest areas, avoids high elevations, extreme temperatures and low precipitation | de Oliveira (1998); Caso et al. (2015) | npp, elevation, bio7, bio15 |
| *Leopardus pardalis* (**ocelot**) | associated with dense forest areas | Murray & Gardner (1997); de Oliveira et al. (2010) | npp, tree, bio10, bio17 |
| *Leopardus wiedii*  (**margay**) | associated with forest areas, avoids high temperatures | de Oliveira (1998b) | npp, nontree, bio7, bio10 |
| *Nasua nasua*  (**coati**) | associated with forest areas | Gompper & Decker (1998) | nontree, npp, bio10, bio13 |
| *Cerdocyon thous*  (**crab-eating fox**) | associated with open and urban areas, avoids dense forest | Tchaicka et al. (2007); Machado & Hingst-Zaher (2009) | urban, tree, bio3, bio4 |
| *Chrysocyon brachyurus* (**maned wolf**) | associated with open grassland areas, avoids dense forest | Dietz (1985); Queirolo et al. (2011) | elev, grass, bio2, bio14 |
| *Eira barbara*  (**tayra**) | rarely found outside forest areas | Presley (2000) | npp, nontree, bio10, bio17 |
| *Pteronura brasiliensis*  (**giant otter**) | associated with wetlands and forest areas | Noonan et al. (2017) | wetland, woodysavanna, bio3, bio5 |

Table A.4. Model evaluation for the presence-absence data measured by Tjur's R^2^ and AUC.

| **Species** | **Tjur’s R^2^** | **AUC** |
| --- | --- | --- |
| *Herpailurus yagouaroundi* | 0.358 | 0.755 |
| *Leopardus pardalis* | 0.272 | 0.66 |
| *Leopardus wiedii* | 0.377 | 0.741 |
| *Nasua nasua* | 0.233 | 0.64 |
| *Cerdocyon thous* | 0.274 | 0.705 |
| *Chrysocyon brachyurus* | 0.237 | 0.666 |
| *Eira barbara* | 0.294 | 0.666 |
| *Pteronura brasiliensis* | 0.15 | 0.714 |

**
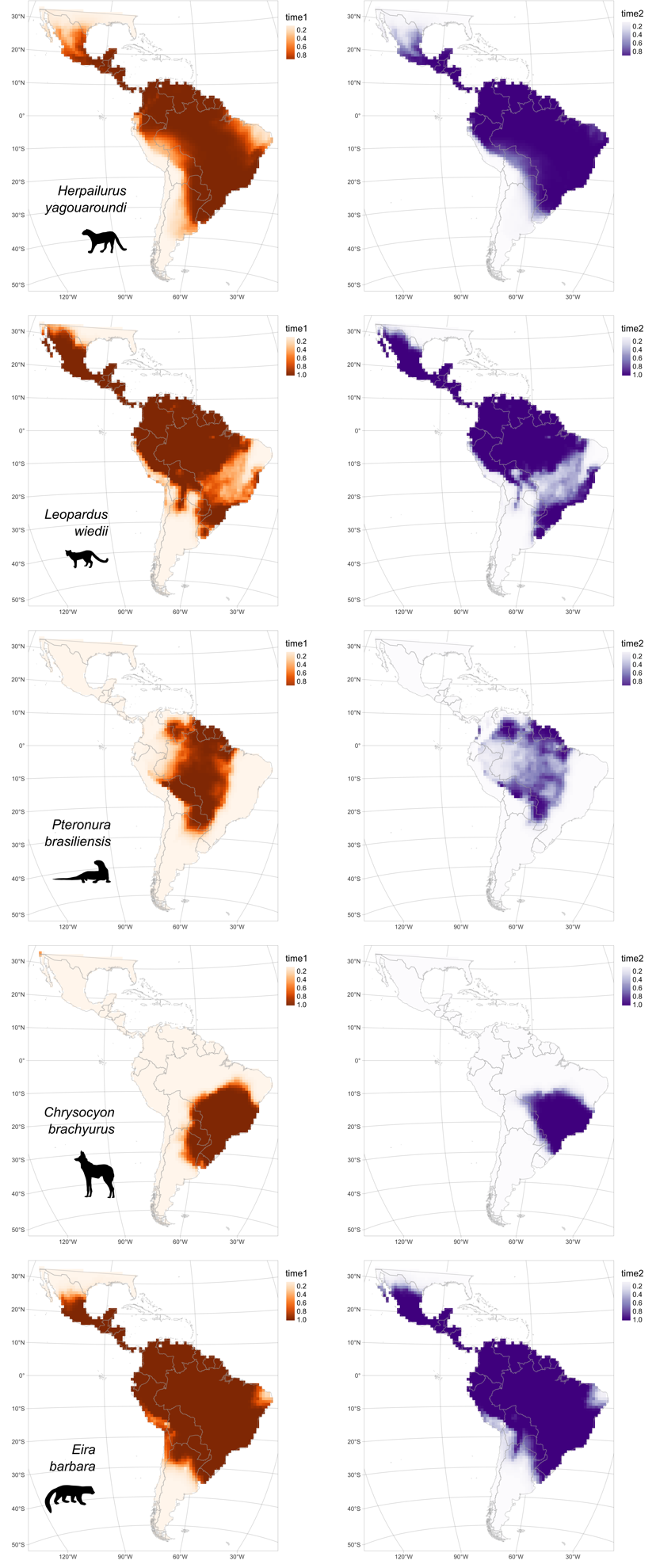
**

**Figure A.1.** Predicted occupancy probability of the species at the two time periods (2000 to 2013 and 2014 to 2021).


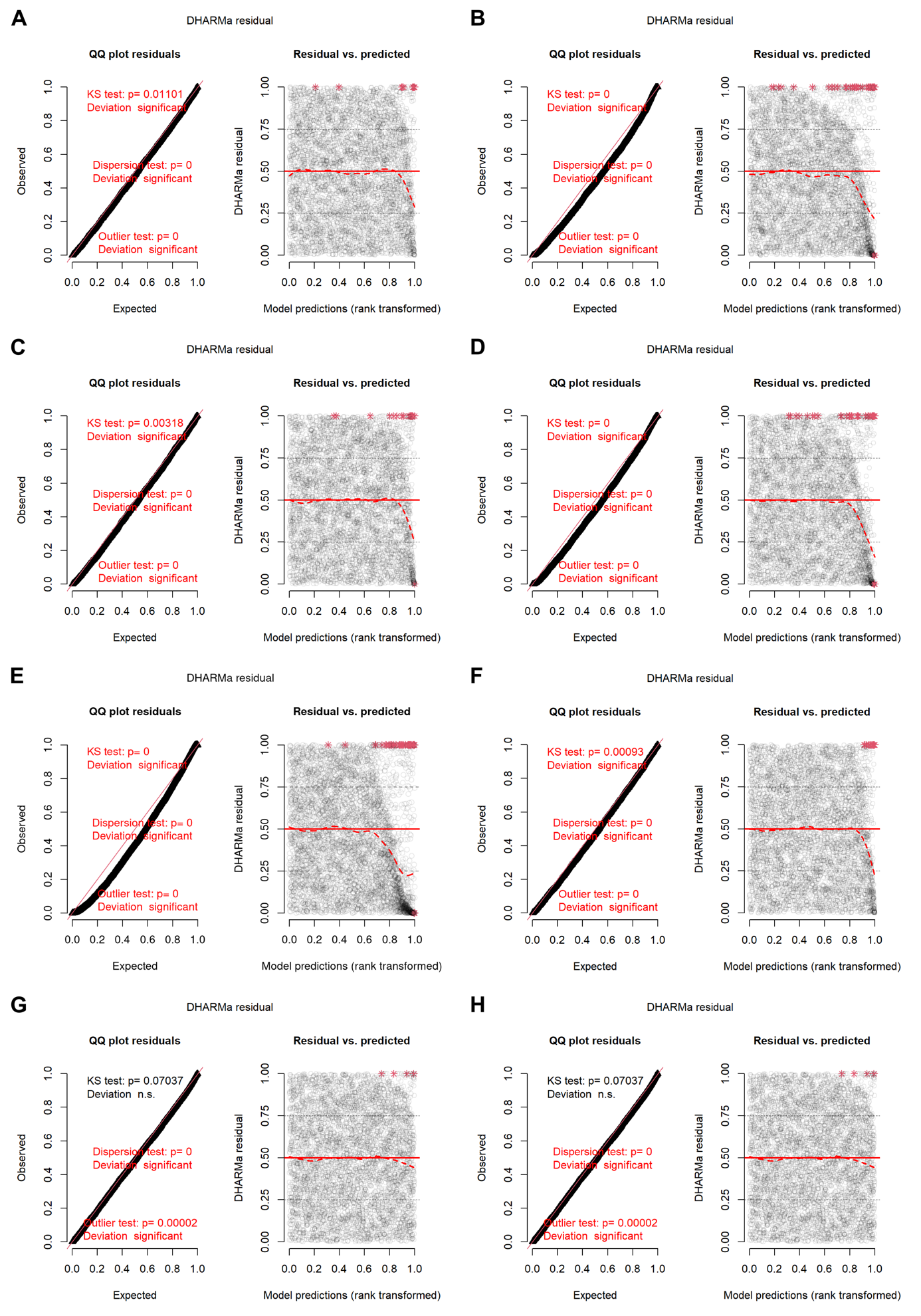


Figure A.2. Model evaluation for the presence-only data assessed. Residual diagnostics fit using the ‘DHARMa’ package (Hartig 2022). (A) *Herpailurus yagouaroundi*, (B) *Leopardus pardalis*, (C) *Leopardus wiedii*, (D) *Nasua nasua*, (E) *Cerdocyon thous*, (F) *Chrysocyon brachyurus*, (G) *Eira barbara*, and (H) *Pteronura brasiliensis*.

**References**

Caso A, de Oliveira T, Carvajal S. 2015. Herpailurus yagouaroundi. The IUCN Red List of Threatened Species 2015: e.T9948A50653167. Page 13. Available from https://www.iucnredlist.org/species/pdf/50653167.

de Oliveira TG. 1998a. Herpailurus yagouaroundi. Mammalian Species:1–6. Available from https://doi.org/10.2307/3504500 (accessed November 13, 2023).

de Oliveira TG. 1998b. Leopardus wiedii. Mammalian Species:1–6. Available from https://doi.org/10.2307/3504400 (accessed November 13, 2023).

de Oliveira TG, Tortato MA, Silveira L, Kasper CB, Mazim FD, Lucherini M, Jácomo AT, Soares JBG, Marques RV, Sunquist M. 2010. Ocelot ecology and its effect on the small-felid guild in the lowland neotropics. Pages 559–580 The biology and conservation of wild felids. Oxford University Press, Oxford.

Dietz JM. 1985. Chrysocyon brachyurus. Mammalian Species:1–4. Available from https://doi.org/10.2307/3503796 (accessed November 13, 2023).

DiMiceli C, Carroll M, Sohlberg R, Kim D, Kelly M, Townshend J. 2015. MOD44B MODIS/Terra Vegetation Continuous Fields Yearly L3 Global 250m SIN Grid V006. NASA EOSDIS Land Processes DAAC.

Fick SE, Hijmans RJ. 2017. WorldClim 2: new 1-km spatial resolution climate surfaces for global land areas. International Journal of Climatology **37**:4302–4315. Available from https://rmets.onlinelibrary.wiley.com/doi/abs/10.1002/joc.5086.

Friedl M, Sulla-Menashe D. 2019. MCD12Q1 MODIS/Terra+Aqua Land Cover Type Yearly L3 Global 500m SIN Grid V006. NASA EOSDIS Land Processes DAAC.

Gompper ME, Decker DM. 1998. Nasua nasua. Mammalian Species:1–9. Available from https://doi.org/10.2307/3504444 (accessed November 13, 2023).

Hartig F. 2022. DHARMa: Residual Diagnostics for Hierarchical (Multi-Level / Mixed) Regression Models. Available from https://CRAN.R-project.org/package=DHARMa.

Machado F de A, Hingst-Zaher E. 2009. Investigating South American biogeographic history using patterns of skull shape variation on Cerdocyon thous (Mammalia: Canidae). Biological Journal of the Linnean Society **98**:77–84. Available from https://doi.org/10.1111/j.1095-8312.2009.01274.x (accessed May 17, 2023).

Murray JL, Gardner GL. 1997. Leopardus pardalis. Mammalian Species:1–10. Available from https://doi.org/10.2307/3504082 (accessed November 13, 2023).

Nagy-Reis M et al. 2020. NEOTROPICAL CARNIVORES: a data set on carnivore distribution in the Neotropics. Ecology **101**:e03128. John Wiley & Sons, Ltd. Available from https://doi.org/10.1002/ecy.3128 (accessed February 24, 2022).

Noonan P, Prout S, Hayssen V. 2017. Pteronura brasiliensis (Carnivora: Mustelidae). Mammalian Species **49**:97–108. Available from https://doi.org/10.1093/mspecies/sex012 (accessed November 13, 2023).

Presley SJ. 2000. Eira barbara. Mammalian Species **2000**:1–6. American Society of Mammalogists. Available from https://bioone.org/journals/mammalian-species/volume-2000/issue-636/1545-1410_2000_636_0001_EB_2.0.CO_2/Eira-barbara/10.1644/1545-1410(2000)636<0001:EB>2.0.CO;2.full (accessed July 4, 2023).

Queirolo D, Moreira JR, Soler L, Emmons LH, Rodrigues FHG, Pautasso AA, Cartes JL, Salvatori V. 2011. Historical and current range of the Near Threatened maned wolf Chrysocyon brachyurus in South America. Oryx **45**:296–303.

Running S, Zhao M. 2019. MOD17A3HGF MODIS/Terra Net Primary Production Gap-Filled Yearly L4 Global 500 m SIN Grid V006. NASA EOSDIS Land Processes DAAC.

Rushing CS, Royle JA, Ziolkowski DJ, Pardieck KL. 2019. Modeling spatially and temporally complex range dynamics when detection is imperfect. Scientific Reports **9**:12805. Available from https://doi.org/10.1038/s41598-019-48851-5.

Tchaicka L, Eizirik E, De Oliveira TG, Cândido Jr JF, Freitas TRO. 2007. Phylogeography and population history of the crab-eating fox (Cerdocyon thous). Molecular Ecology **16**:819–838. Available from https://onlinelibrary.wiley.com/doi/abs/10.1111/j.1365-294X.2006.03185.x (accessed May 17, 2023).

Wood SN. 2003. Thin-plate regression splines. Journal of the Royal Statistical Society (B) **65**:95–114.

Wood SN. 2017. Generalized Additive Models: An Introduction with R, 2nd edition. Chapman and Hall/CRC, New York. Available from https://doi.org/10.1201/9781315370279.
