# Supplementary material for: A continental-wide decline of occupancy and diversity in five Neotropical carnivores
Source: Glob Ecol Conserv. 2024 Nov;55:e03226. doi: 10.1016/j.gecco.2024.e03226 (PMC11513410; doi:10.1016/j.gecco.2024.e03226)
Supplement: Supplementary file 2 — Appendix B Supplementary material [file mmc2.docx]

Appendix B

Full model description

**1 Point pattern intensity**

The model assumes that a species’ distribution can be described by a continuous point pattern intensity across Latin America. To create the point pattern intensity, we used design matrices ($X_{PA}$ and $X_{PO}$) that contained as many columns as the fitted model has parameters; an intercept, environmental covariates, the distance to the expert range, and the spline bases (varying number for each species), and as many rows as blobs or grid-cells respectively.

When the design matrices are multiplied by the vector of parametric effects ($\mathbf{b}$), they yield the linear predictors ($\eta_{PA}$ and $\eta_{PO}$), i.e., the expected point pattern intensity, given the values of all explanatory variables in the model:

$\boldsymbol{\eta}_{\mathbf{PA}}=\mathbf{X}_{\mathbf{PA}}\times\mathbf{b}$ (1)

$\boldsymbol{\eta}_{\mathbf{PO}}=\mathbf{X}_{\mathbf{PO}}\times\mathbf{b}$ (2)

Note here that $\mathbf{b}$ is the same in both Equation 1 and Equation 2; this is the central part of the model that connects the PO and PA data and allows the calculation of the joint likelihood. For the $\mathbf{b}$ parameters, we used Gaussian priors with 0 mean and SD of 10, i.e., $b_{r}\sim\text{Normal}\left( 0,0.01 \right)$, where $r\in1:n_{par}$ (total number of parameters in $\mathbf{b}$).

**2 Smoothing splines**

We used thin plate regression splines (Wood, 2003) to model the spatial structure in the distribution that was not accounted for by the environmental covariates. These spatial splines give our model the flexibility to predict absences in otherwise suitable environments, which can happen due to dispersal limits, demographic stochasticity, or biotic interactions. In other words, the splines enable us to model the realised distribution in each time period, i.e., the actual distribution affected by its ecological preferences and factors such as dispersal limitation, and not just the fundamental distribution given solely by the environmental conditions (Rushing et al., 2019). For instance, over time it can indeed happen that an area with unsuitable static climate may become suitable, which is not captured by our static predictors. This is where the splines do their job, as they naturally track these deviations from the average. Because of this, the splines can provide hints about the types of environmental variation responsible for the dynamics.

We fit a different spatial spline for each time period. First, we generate $k$ spline basis variables prior to the model fitting, using the jagam function from the ‘mgcv’ package (Wood, 2017). The number of $k$ varied for each species, between 9 and 11. These variables are then part of the $X_{PA}$ and $X_{PO}$ matrices, and they have their own corresponding coefficients in the $\mathbf{b}$ vector. These coefficients have their own multivariate normal prior, specified using smoothing penalty matrices and smoothing parameters. Priors for time1 and time2 are:

$\sigma_{time1}=S_{time1_{1:n.spl,1:n.spl}}\times\gamma_{1}+S_{time1_{1:n.spl,n.spl+1:n.spl\times2}}\times\gamma_{2}$ (3)

$b_{n.par+1:n.spl+n.par}\sim\text{Normal}\left( Z_{n.par+1:n.spl+n.par},\sigma_{time1} \right)$ (4)

$\sigma_{time2}=S_{time2_{1:n.spl,1:n.spl}}\times\gamma_{3}+S_{time2_{1:n.spl,n.spl+1:n.spl\times2}}\times\gamma_{4}$ (5)

$b_{n.X-n.spl+1:n.X}\sim\text{Normal}\left( Z_{n.X-n.spl+1:n.X},\sigma_{time2} \right)$ (6)

For more details about this, refer to the help of the jagam R function.

**3 Modelling presence-absence data**

Letting $y_{PA_{i}}$ refer to the observed presence (=1) or absence (=0) value in each $i$-th blob for the first or second period, we modelled the blob-specific probability of presence ($\psi_{i}$) as a function of the fixed effects of the presence-absence linear predictor ($\eta_{PA_{i}}$) and sampling effort ($\text{effort}_{i}$, i.e., number of camera trap days), and the logarithm of the area of each blob in m^2^ ($\text{area}_{PA_{i}}$) as an offset term,

$cloglog\left( \psi_{i} \right)=\eta_{PA_{i}}+log\left( \text{area}_{PA_{i}} \right)+\beta\times log\left( \text{effort}_{i} \right)$ (7)

where the index $i$ identifies blobs. Prior distribution of $\beta$ was $\beta\sim\text{Normal}\left( 0,0.01 \right)$, i.e., Gaussian prior with mean zero and standard deviation of 10. This prior distribution is sufficiently uninformative given the scale of the predictors (scaled to 0 mean and SD=1), while also not too wide to hinder MCMC convergence.

The state of this variable follows a Bernoulli distribution with mean $\psi_{i}$ where $y_{PA_{i}}$ is the observed data,

$y_{PA_{i}}\sim\text{Bernoulli}\left( \psi_{i} \right)$ (8)

**4 Modelling presence-only data**

We assume that the spatial distribution of individuals may be modelled using a Poisson point process. In our model, the true intensity (i.e., the mean number of presence points per grid-cell) for the species in each grid-cell $j$ is denoted as $\nu_{j}$. We modelled it as a function of the presence-only linear predictor ($\eta_{PO_{j}}$) by the logarithm of the area of each grid-cell in m2 ($\text{area}_{PO_{j}}$).

$\log\left( \nu_{j} \right)=\eta_{PO_{j}}+log\text{area}_{PO_{j}}$ (9)

where $j$ denotes a grid-cell.

To model the thinning of the true intensity, we calculated the cell-specific probability of retaining/observing a point ($P_{ret_{j}}$) as a decaying exponential function with a random intercept $\alpha_{0}\left[ \text{country}_{j} \right]$ for each $country$-th country and a fixed slope $\alpha_{1}$ for grid-cell accessibility ($\text{acce}_{j}$),

$P_{ret_{j}}=\alpha_{0}\left[ \text{country}_{j} \right]\times\exp^{-\alpha_{1}\times\text{acce}_{j}}$ (10)

The prior distribution for $\alpha_{0}\left[ \text{country}_{j} \right]$ was Beta distribution with shape parameters equal to one, i.e., $\alpha_{0}\left[ \text{country}_{j} \right]\sim\text{Beta}\left( 1,1 \right)$ where $c\in1:n_{\text{country}}$, (total number of countries). This is a flat prior that gives equal probability density to every value between 0 and 1. The prior for the slope of the distance decay $\alpha_{1}$ was a Gamma distribution with shape 0.5 and scale 0.05, i.e., $\alpha_{1}\sim\text{Gamma}\left( 0.5,0.05 \right)$. This is a weakly informative prior that is skewed to take small values; it is wide enough to be effectively non-informative given the meaningful parameter values, but not too wide to hinder MCMC convergence.

Finally, we calculated the thinned intensity per grid-cell ($\lambda_{j}$) as the product of the true intensity ($\nu_{j}$) times the probability of retaining a point per grid-cell ($P_{ret_{j}}$), for the both time periods, and included a $\text{global.effort}_{j}$ multiplier, for the second period. With this, we calibrate the estimated number of records per area by the overall sampling effort (measured by the ratio of records between time1 and time2 for all carnivores’ data in GBIF).

$\lambda_{j}=\left\{ \begin{matrix} \nu_{j}\times P_{ret_{j}} & \text{if }j>n_{PO}/2\text{(time}_{1}\text{)} \\ \nu_{j}\times P_{ret_{j}}\times\text{global.effort}_{j} & \text{otherwise}\text{(time}_{2}\text{)} \end{matrix} \right.$ (11)

The state of this variable follows a Poisson distribution with mean $\lambda_{j}$ where $y_{PO_{j}}$ is the observed data,

$y_{PO_{j}}\sim\text{Poisson}\left( \lambda_{j} \right)$ (12)

**5 Predictions**

To predict the probability of occurrence of the species in the two time periods, we used the linear predictor $\boldsymbol{\eta}_{\mathbf{pred}}$, as

$\boldsymbol{\eta}_{\mathbf{pred}}=\mathbf{X}_{\mathbf{PO}}\times\mathbf{b}$ (13)

The detection probability ($P_{pred_{j}}$) was modelled for each grid-cell $j$ with the logarithm of the area ($\text{area}_{PO_{j}}$) as an offset term.

$cloglog\left( P_{pred_{j}} \right)=\eta_{pred_{j}}+log\left( \text{area}_{PO_{j}} \right)$ (14)

**6 Derived quantities**

Finally, as derived outputs of the model, we calculated the area of the species range for the first period ($A_{time1}$) and the second period ($A_{time2}$) with $n_{PO}$ as the total number of grid cells for both periods together,

$A_{time1}=\sum\eta_{pred_{j}} \text{where}j\in1:n_{PO}/2$ (15)

$A_{time2}=\sum\eta_{pred_{j}} \text{where}j\in n_{PO}/2:n_{PO}$ (16)

and the difference in the area (in number of 100×100km grid-cells) for both time periods ($\Delta A$):

$\Delta A=A_{time2}-A_{time1}$ (17)

$\Delta SD_{j}=P_{pred_{\left( n_{PO}/2 \right)+j}}-P_{pred_{j}}$ (18)

**References**

Rushing, C. S., Royle, J. A., Ziolkowski, D. J., & Pardieck, K. L. (2019). Modeling spatially and temporally complex range dynamics when detection is imperfect. *Scientific Reports*, *9*(1), 12805. https://doi.org/10.1038/s41598-019-48851-5

Wood, S. N. (2003). Thin-plate regression splines. *Journal of the Royal Statistical Society (B)*, *65*(1), 95–114. https://doi.org/10.1111/1467-9868.00374

Wood, S. N. (2017). *Generalized Additive Models: An Introduction with R* (2nd ed.). Chapman and Hall/CRC. https://doi.org/10.1201/9781315370279
